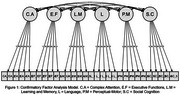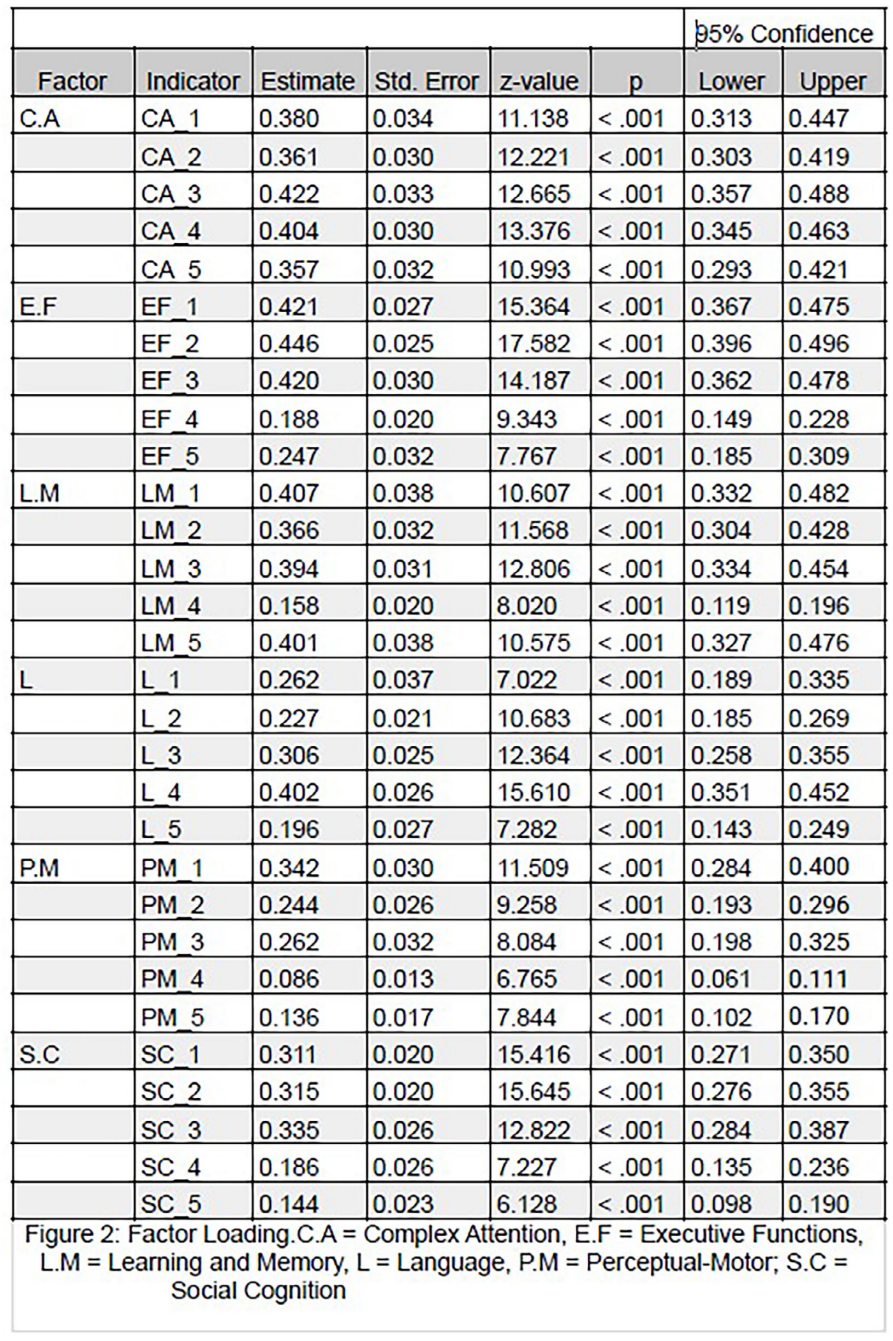# Confirmatory Factor Analysis of the Participant Version of Cognitive Domains and Functional Assessment Questionnaire

**DOI:** 10.1002/alz70857_103756

**Published:** 2025-12-25

**Authors:** Aline Siqueira de Souza, João Vitor da Silva Viana, Isabelle de Aguiar Maia, Caio Peixoto Tavares, Giovanna Correia Pereira Moro, Guilherme Vieira Rodrigues Da Costa, Alberto Fonseca Lopes Rodrigues, Gabriela Tomé Oliveira Engelmann, Marco Aurélio Romano‐Silva, Jonas Jardim de Paula, Maria Aparecida Camargos Bicalho, Bernardo de Mattos Viana

**Affiliations:** ^1^ Sciences Applied to Adult Health Postgraduate Program, School of Medicine, Universidade Federal de Minas Gerais (UFMG), Belo Horizonte, Minas Gerais, Brazil; ^2^ Older Adult's Psychiatry and Psychology Extension Program (PROEPSI), School of Medicine, Universidade Federal de Minas Gerais (UFMG), Belo Horizonte, Minas Gerais, Brazil; ^3^ Cog‐Aging Research Group, Universidade Federal de Minas Gerais (UFMG), Belo Horizonte, Minas Gerais, Brazil; ^4^ Federal University of Minas Gerais, Belo Horizonte, Minas Gerais, Brazil; ^5^ Cog‐Aging Research Group, Belo Horizonte, Minas Gerais, Brazil; ^6^ Undergraduate Medicine, Faculty of Medicine, Universidade Federal de Minas Gerais (UFMG), Belo Horizonte, Minas Gerais, Brazil; ^7^ Older Adult's Psychiatry and Psychology Extension Program I Federal University of Minas Gerais, Belo Horizonte, Minas Gerais, Brazil; ^8^ Undergraduate Medicine, School of Medicine, Universidade Federal de Minas Gerais (UFMG), Belo Horizonte, Minas Gerais, Brazil; ^9^ Universidade Federal de Minas Gerais, Belo Horizonte, Brazil; ^10^ Jenny de Andrade Faria Institute – Outpatient Reference Center for the Elderly, Universidade Federal de Minas Gerais (UFMG), Belo Horizonte, Minas Gerais, Brazil; ^11^ Molecular Medicine Program, School of Medicine, Federal University of Minas Gerais, Belo Horizonte, Minas Gerais, Brazil; ^12^ Molecular Medicine Postgraduate Program, School of Medicine, Universidade Federal de Minas Gerais (UFMG), Belo Horizonte, Minas Gerais, Brazil; ^13^ Department of Psychiatry, School of Medicine, Federal University of Minas Gerais, Belo Horizonte, Minas Gerais, Brazil; ^14^ Neurotec R National Institute of Science and Technology (INCT‐Neurotec R), Faculty of Medicine, Universidade Federal de Minas Gerais (UFMG), Belo Horizonte, Minas Gerais, Brazil; ^15^ Universidade Federal de Minas Gerais, Belo Horizonte, Minas Gerais, Brazil; ^16^ INCT – NeuroTecR and CTMM, Belo Horizonte, Minas Gerais, Brazil; ^17^ Older Adult's Psychiatry and Psychology Extension Program I Federal University of Minas Gerais, Belo Horizonte, MG, Brazil; ^18^ Geriatrics and Gerontology Center Clinical Hospital of Universidade Federal de Minas Gerais, Belo Horizonte, Minas Gerais, Brazil; ^19^ National Institute of Science and Technology Neurotec R (INCT‐MM), Belo Horizonte, Minas Gerais, Brazil; ^20^ Department of Internal Medicine, School of Medicine, Federal University of Minas gerais, Belo Horizonte, Minas Gerais, Brazil; ^21^ Department of Clinical Medicine, Faculty of Medicine, Universidade Federal de Minas Gerais (UFMG), Belo Horizonte, Minas Gerais, Brazil; ^22^ Hospital das Clínicas da UFMG, University Hospital, Universidade Federal de Minas Gerais (UFMG), Belo Horizonte, Minas Gerais, Brazil; ^23^ National Institute of Science and Technology (INCT‐Neurotec R), Faculty of Medicine, Federal University of Minas Gerais, Belo Horizonte, Minas Gerais, Brazil; ^24^ Older Adult's Psychiatry and Psychology Extension Program Federal University of Minas Gerais, Belo Horizonte, Minas Gerais, Brazil; ^25^ Geriatrics and Gerontology Center Clinical Hospital of University of Minas Gerais, Belo Horizonte, Minas Gerais, Brazil; ^26^ Jenny de Andrade Faria Institute – Outpatient Reference Center for the Elderly, Universidade Federal de Minas Gerais (UFMG), Belo Horizonte, Minas Gerais, Brazil

## Abstract

**Background:**

The Cognitive Domains and Functional Assessment Questionnaire (CDFAQ) assesses cognitive and functional decline based on the DSM‐5 criteria for Neurocognitive Disorders. Its accuracy has been assessed, and it has been translated and adapted into English. The participant version (CDFAQ‐PV) is a 30‐item questionnaire that assesses six cognitive domains, based on the DSM‐5, with five items each: Complex Attention (CA), Executive Functions (EF), Learning and Memory (LM), Language (L), Perceptual‐Motor (PM) and Social Cognition (SC). Although the informant version (CDFAQ‐IV) has already been assessed by factor analysis with convergence to the DSM‐5 cognitive domains, this assessment hasn’t been done yet for the participant version.

**Objective:**

To perform a Confirmatory Factor Analysis of the CDFAQ‐PV to assess the six‐factor cognitive domain model.

**Methods:**

Older adults and their informants were invited to participate in this study. The CDFAQ‐PV was applied in 377 older adults. We used the JASP for a Confirmatory Factor Analysis based on Lavaan R Packages. The confirmatory factor analysis was run with a manual six‐factor model. This study was approved by the ethics committee of UFMG.

**Results:**

Confirmatory factor analysis model fitness was significant with X2(p < .001), with standardized root mean square residual (SRMR) .065 (accepted < .08), and with the goodness of fit index (GFI) .983 (accepted > .9). However, the root mean square error of approximation (RMSEA) was over the accepted values .073 (accepted < .06), as well as the comparative fit index CFI that was .792 under the accepted cutoff (accepted > .9).

**Conclusion:**

The six‐factor model of the showed a good fit for three parameters, and negative for two. These results point to a convergence of the CDFAQ‐PV and CDFAQ‐IV, as well to the DSM‐5 cognitive domains. These are still preliminary results and we aim to increase our sample to further assess the confirmatory factor analysis.